# Dr. Williams and the Association of Faculties

**Published:** 1896-08

**Authors:** 


### DR. WILLIAMS AND THE ASSOCIATION OF FACULTIES.

Dr. Williams in his recent series of valuable papers, running through several numbers of the *Dental Cosmos*, takes occasion, in a foot-note, page 464 of the June number, to criticise the action of that body in indorsing Dr. Bödecker's book. We quote in part this note:

"Professor Heitzmann and his pupils, particularly Drs. Bödecker and Abbott, have during the past fifteen years laid great stress upon their claims to the discovery of a minute structure of living organic matter in enamel. . . . Those of us who have long been familiar with these errors did not attach any special importance to the 'vain imaginings' of the Heitzmann School until these errors received the indorsement of the National Association of Dental Faculties. That action removed the entire question beyond the realm of personal controversy. It has now become a matter of national importance to the dental profession in America, and the question is, Are dental students in the United States to be taught theories of dental histology which are only mentioned by scientific men of standing, the world over, with contempt or ridicule? Let it be distinctly understood that it is not a matter of opinion, but of fact, and it is facts which the National Association of Dental Faculties of the United States have to face in considering whether they will withdraw their indorsement of a book scarcely a page of the histological work of which is free from some statement that the dental student will not have to unlearn."

It is evident from this quotation that Dr. Williams does not understand the action of the Association of Faculties, and, in future, it might be well to familiarize himself with its methods of work before he publicly holds up a body of earnest workers to wholesale condemnation.

The indorsement by that body is given after a careful examination by a standing committee appointed for that purpose. The recommendation is not understood to carry with it any indorsement of the peculiar views held by an author, or is its action binding in this respect upon the colleges constituting its membership; and, further, the recommendation does not go so far as to even suggest that any work so recommended shall be used as a text-book in the colleges, although that word has been used, but it simply means that an author has published a book worthy of consideration by men engaged in teaching, and does not carry any force beyond that statement. If the ideas conveyed therein are in accord with those held by any given faculty they may be taught, or if in opposition they may be antagonized. The Association of Faculties has nothing directly to do with the teachings of Dr. Williams, Dr. Bödecker, or Dr. Abbott, but leaves the matter to the judgment of its individual members. It is not probable that any well-organized school would accept the productions of either of these gentlemen as a finality or adopt them as text-books, but would regard them as valuable works of reference, a place they very properly hold in dental literature.

The position held by the National Association of Dental Faculties is a dignified and proper one, and is not likely to be changed by any criticism come from what quarter it may.

